# Indian research on acute organic brain syndrome: Delirium

**DOI:** 10.4103/0019-5545.69226

**Published:** 2010-01

**Authors:** Charles Pinto

**Affiliations:** Department of Psychiatry, BYL Nair Hospital and TN Medical College, Mumbai, India

**Keywords:** Delirium, Indian, studies

## Abstract

Delirium, though quite often referred to psychiatrists for management, does not find many takers for analysis, research and publications. Acute in onset, multiplicity of etiology and manifestations, high risk of mortality delirium is very rewarding in proper management and outcome. Delirium has a limited agenda on teaching programs, research protocols and therapeutic strategies. There is a dearth of Indian studies both in international and national scientific literature. This annotation is based on a Medline search for “delirium India” on Pubmed, which resulted in 54 articles. A search in Indian Journal of Psychiatry for “delirium” resulted in 38 published articles, “delirium tremens” showed up only five articles. The articles are primarily from the Indian Journal of Psychiatry with cross reference to articles on Pubmed or Google search on Indian studies and a few international studies

## INTRODUCTION

Delirium is an acute organic brain syndrome with increased morbidity and mortality. It is derived from Latin, meaning “off the track”. Sutton described delirium tremens. Delirium is a complex neuropsychiatric syndrome presenting primarily with disturbances of cognition, perception and sensorium, alertness, sleep/wake cycle, and psychomotor behavior in the context of a medical etiology. The presentation can be quite variable among patients and even within a given patient because of its waxing and waning course. This variability and overlap with other psychiatric syndromes has led to substantial under recognition and under treatment in clinical settings.[1] It is a transient cognitive impairment syndrome but an emergency as it has a favorable outcome if diagnosed and treated early. Delirium occurs in 30% of hospitalized patients and is associated with prolonged hospital stay and increased morbidity and mortality. Delirium is a disturbance of consciousness characterized by acute onset, rapid fluctuations in mental status and impaired cognitive functioning. The patient’s ability to receive, process, store and recall information is strikingly impaired. The patient may be agitated or lethargic. The mnemonic for the differential diagnosis I WATCH DEATH (Infections, Withdrawal, Acute metabolic encephalopathy, Trauma, central nervous system (CNS) pathology, hypoxia, deficiencies, endocrine disorders, acute vascular insufficiency, toxins and drugs, heavy metals) points to the fact that delirium may be the symptom of a serious underlying life-threatening disorder. Childhood delirium has a different course and symptom profile than adult and geriatric delirium.[2] Adult and geriatric delirium differs only in the severity of cognitive symptoms.

The annotations on Acute Organic Brain Syndrome Delirium are conveniently described as they have been published in the Indian Journal of Psychiatry as clinical studies/research/brief communication; case histories/reports; editorials, reviews, invited articles; Presidential addresses/orations and poster/paper presentations, viewpoint, symposia. The reference and relevance of delirium in the article is highlighted with the authors comments in italics

## CLINICAL/RESEARCH STUDIES

### Original research

A study by Pawar *et al*. on “Cognitive and emotional effects of renal transplantation”[3] notes that Organic brain syndromes such as delirium and dementia are also common in these patients. However, their study revealed an 86.7% prevalence of depression in ESRD patients as compared to 56.7% in post renal transplant patients and an analysis of neurocognitive functions on LNNB did not reveal any significant impairment. Hence they found no delirium or dementia in their study.

Prasad *et al*. in their original research article “Clinical practice with antidementia and antipsychotic drugs: Audit from a geriatric clinic in India”[4] found donepezil to be the most commonly prescribed antidementia drug and quetiapine to be the most commonly used antipsychotic in a tertiary care geriatric clinic, in a developing country. Behavioral and psychological symptoms of dementia (BPSD), are an integral part of the dementia syndrome but the management of BPSD requires clarification of target symptoms, ruling out delirium and comorbid major psychiatric diagnoses, and creatively addressing possible social, environmental, or behavioral remedies along with pharmacotherapy. Delirium here was exclusion for BPSD before treatment

Das *et al*. in their study “Intentional self-harm seen in psychiatric referrals in a tertiary care hospital”[5] found that majority of the subjects (78%) had a precipitating event prior to the intentional self-harm, most common of which was interpersonal problems with family members other than spouse (38%), followed by interpersonal problems with spouse (16.9%). The most common method of self-harm was consumption of insecticides (44.6%), followed by use of corrosives (17.5%) and use of psychotropic drugs (12.7%). About 17% of patients had harmed themselves after consuming alcohol. Out of the psychotropics, sedative-hypnotic group was the commonly used drug for overdose. Use of insecticides was almost equal in both genders (48% for males and 51% for females). However, more fatal methods like stabbing self with a sharp instrument, guns shot, jumping in front of train were observed only in males (*n* = 8, 4.8%). In more than half of the cases (56.6%), intentional self-harm did not lead to any severe medical complications. Sixteen per cent of the patients developed delirium, 10% developed post corrosive esophageal ulceration and 3.6% developed hypoxic ischemic encephalopathy as a complication to the methods used for intentional self-harm. Delirium was described as one of the complications of the method of self harm.

Kunigiri *et al*. in their study “MRI T_2_ relaxometry of brain regions and cognitive dysfunction following electroconvulsive therapy”[6] examined alteration in magnetic resonance imaging (MRI) T_2_ relaxation time, a measure of brain edema, and its relation to therapeutic efficacy, orientation and memory impairment with ECT. In their methodology, two patients each had emergent delirium after the second ECT (both BLECT patients) and the fifth ECT (both ULECT patients). It was managed by thiopentone (50-75 mg) administered intravenously. Their findings suggest that ECT does not produce demonstrable change acutely in brain parenchyma detectable by MRI scans. Delirium was a treatment emergent event during ECT in two patients each out of 15 patients in the sample in the methodology, resolved with thiopentone and had no effect on the findings of the study.

Tirupati *et al*. in their study of “Cognitive decline in elderly medical and surgical inpatients”[7] point out that impairment in cognitive function increases with age. It has been seen that nearly 50% of people admitted in the medical and surgical wards of a general hospital are over 65 years of age. Delirium and dementia are the two common syndromes of general cognitive decline. Delirium occurs in 10-61% and dementia in 14-61% of elderly inpatients in general hospitals. They were associated with an increased morbidity and mortality, length of hospitalization, cost of care and caregiver stress. Medical teams often fail to diagnose cognitive decline in elderly inpatients in their literature review. The paper reports on the prevalence of cognitive decline in patient’s ≥60 years of age admitted to a community general hospital for medical or surgical treatment. They selected 130 patients comprising 85 men and 45 women for the study. The Mini Mental State Examination (MMSE) was used to identify subjects with cognitive dysfunction. Information on recent history of decline in memory, thinking and reasoning ability was elicited from the subject and the key informant using the informant and subjective versions of the Global Rating of Memory Decline (GRMD) and Global Rating of Intellectual Decline (GRID). The GRMD was used to assess if the patient had any problem with memory compared to the past (answered as Yes, Sometimes and No) and, if yes, whether the decline in memory interfered in any way with day-to-day life (answered as Yes, No, Do not know). The GRID was used to ask similar questions about any change in the patient’s ability to think and reason.

The prevalence of cognitive decline as defined by MMSE in the hospitalized elderly population in this study was 41.5. They felt that screening to detect cognitive decline in elderly inpatients could facilitate investigation to identify reversible causes of dementia and plan appropriate treatment to improve the outcome. However, no case of delirium is mentioned in their findings though cognitive decline is mentioned which was the primary objective of the study. Also, the instruments used may not be able to detect delirium.

### Clinical study

Kumar *et al*. in “Inhalant abuse: A clinic-based study published report inhalant abuse/dependence”[8] where consecutive treatment seeking inhalant abuse cases (*n* = 21) were studied for the sociodemographic and clinical profile by using a semi-structured interview schedule concluded that the results depict that easy availability, cheap price, faster onset of action, and a regular high makes inhalant a substance of abuse especially among the urban youth as all 21 cases were using TPEF; one case also sniffed petrol vapors. Two-third (66.66%) subjects reported an immediate intoxication as “kick/high/euphoria/feeling of relaxation” with one or more of the following symptoms: Giddiness (23.8%), unsteadiness or perceptual disturbances (19.0% each), unconsciousness or delirium (14.3% each) and lightheadedness (9.5). One or more of the following withdrawal symptoms were reported by 57.1% cases only: Irritability (23.8%), subjective restlessness (14.3%), observed restlessness (9.5%), and insomnia, tingling sensation all over the body, headache and poor concentration (4.8% each). Co morbid seizure disorder was recorded in 9.5% cases. The recorded comorbid psychiatric disorders included conduct disorder (19%) and schizophrenia (4.8%). They, however, cautioned on excessive generalization of their findings as unwarranted for many reasons; as they focused only on treatment seekers; carried out retrospective chart review; some of their instruments and definitions (e.g., social support, impairment, motivation, and outcome) are study/center specific and untested for reliability; and, findings could be attributable to other substances being used or abused as well. Delirium was still not a single entity in this study.

Avasthi *et al*. in their study “Psychiatric profiles in medical-surgical populations: Need for a focused approach to Consultation-Liaison psychiatry in developing countries”[9] report that in their Psychiatric assessment The frequency distribution of psychiatric diagnosis was organic psychosis (25.5%) non-organic psychosis (11.2%), neurosis(24.8%), ‘other disorders’ (21.5%) and ‘nil psychiatric’ (17%). Among neurotic disorders, depressive neurosis was the commonest subcategory (14.8% of all referrals), while substance abuse disorders (6.7%) and adjustment reaction (5.8%) contributed significantly to the ‘other disorders’. Most of the organic psychosis was of an acute nature. Organic psychosis was common in the geriatric (>60 years) age group. Organic psychosis was common in referrals from Obstetrics and Gynaecology Organic psychosis was frequently diagnosed in those with infective and obstetric and gynecologic conditions. Though there is no mention of Delirium as a group it may be inclusive in Organic psychosis.

Madan *et al*. in their clinical study “Sociodemographic profile and psychiatric morbidity in HIV seropositive defense personnel”[10] had 172 HIV-seropositive subjects in CDC stage II, III and IV compared them with 40-seronegative controls. Heterosexual promiscuous activity was found to be predominant mode of HIV-infection transmission (92.44%). Overall psychiatric morbidity was found in 50% of study groups compared to 10% in controls. The break-up of diagnostic categories as per ICD-10 criteria was - depressive episode 22.9%, anxiety disorder 9.86%, alcohol dependence syndrome 6.39%, delirium 1.16% and cognitive impairment 10.47%. The study highlights that HIV-epidemic and its associated psychiatric morbidity is largely a behavioral problem and hence calls for an active participation of mental health professionals to counteract the challenge posed by it.

The study is one of the earliest to make a separate diagnosis for delirium.

Mattoo *et al*. in “Clinical course of alcohol dependence”[11] describe 47 subjects having alcohol dependence syndrome and the progression of alcohol related milestones in terms of age-at-onset of each milestone. The findings revealed a definable progression with three phases. The early phase, characterized by the absence of any problem, ended with the use of 1/4^th^ bottle of spirit a day, more than once-a-week. The middle phase began with daily drinking, ended with the use of 1 bottle of spirit a day, and was characterized mainly by social problems. The late phase began with the onset of morning drinking, and was characterized by the addition of physical problems.

Correlations between specified milestones: The correlation coefficients between age-at-onset of three drinking dyscontrol milestone i.e. daily, daytime, and morning-drinking, and certain social and physical milestones revealed that the correlations were highly significant (*P* < 0.001) between all the three drinking dyscontrol milestones and job loss, drunken brawl, borrowing money for drinks, absenteeism, blackouts, morning shakes, memory lapses and hospitalization and more significant between delirium tremens and, daytime-, and morning - drinking (*P* < 0.001) than between delirium tremens and daily-drinking (*P* < 0.01).

This study is one of the earliest to evaluate delirium tremens.

Chaudhary *et al*. in “Post Cataractomy delirium: A 2-year prospective study in 221 consecutive inpatients undergoing cataractomy”[12] found the incidence of delirium at 1.8%. While in one case it was due to Anticholinergic toxicity, in the other two there was no organic cause. Sensory deprivation was present in two cases. Post Cataractomy Delirium (PCD) was diagnosed as per Summers and Reich criteria. There were four patients in the first year and none in the second year which was lower than in international studies. They suggested awareness of the problem, avoidance of anticholinergic drugs, avoidance of sensory deprivation with increase in orientation and sensory stimuli may prevent Post Cataractomy Delirium.

Delirium seen in this condition shows multiple etiology and management strategy has been defined.

Bhattacharya *et al*. in their study “Puerperal Psychosis”[13] studied 50 cases of puerperal psychosis where majority were in 15-30 years (80%) of age and from rural area. 76% had infection. They found schizophrenia in 76%, Neurotic reactions 10%, Sub acute delirious state in 10% and depression in 4%. They emphasized the puerperal sepsis etiology.

Delirium not defined clearly and the sub acute nature mentioned, but clearly, is a high referral for this condition.

Khurana *et al*. in their study “Prevalence of Delirium in Geriatric hospitalized general medical population”[14] was carried out within 24 hours after admission and every fourth day thereafter. The assessment was done using MMSE, Confusion Assessment Method (CAM), Delirium Symptom Interview (DSI) and ICD 10 DCR for Delirium. An overall rate of Delirium of 27% was found in the sample of 100 patients. The ‘prevalent’ delirium rate was 19% and the ‘incident’ rate of delirium was 8%. It is observed that the CAM is a useful screening method with high sensitivity for diagnosis of delirium at the bedside.

A comprehensive study on delirium including the rates of delirium but most importantly the screening tool of CAM for delirium

Sabhesan *et al*. in a study “Post Traumatic Hyperactive Delirium”[15] point out that it is a common problem following head injury; 29 patients who were diagnosed with hyperactive delirium were prospectively followed up during their stay in hospital. When these were compared with controls, alcohol dependence was significantly more among the patients than controls. The occurrence of delirium was related to the generalized cerebral disturbances; due to diffuse damage in acceleration injuries and metabolic or post seizure disturbances in contact injuries. A follow up of the patients showed that psychiatric problems were more common among them.

A most distinct type of delirium with implications on etiology and sequelae.

### Brief communication

Sood *et al*. in their study of “Psychiatric morbidity in non-psychiatric geriatric inpatients”[16] had an aim to evaluate the profile of psychiatric disorders in geriatric inpatients. A total of 528 patients (age 65 years and above) admitted to various departments of the teaching hospital were submitted to Psychiatric assessment on the basis of psycho geriatric assessment scales (PAS) and present state examination (PSE-ninth edition, 1974). The ICD-10 criteria were used for psychiatric diagnoses. General medical conditions were diagnosed by consultants of the respective departments. The obtained data analyzed of the 528 patients, had 260 (49%) with psychiatric co-morbidity. The most common psychiatric disorder was depression (25.94%), followed by adjustment disorders (11%), anxiety disorders (4.54%), dementias (3.6%), delirium (3%), bipolar disorders (0.8%), and substance-related disorders (0.4%). In the present study, delirium was found in 3% and most of the patients (1.13%) were suffering from neurological conditions, closely followed by endocrinological conditions (0.94%). The above findings emphasized the importance of consultation-liaison psychiatry, especially in geriatric patients.

The study clearly emphasizes the need for CL Psychiatry for delirium and also the right tool for assessment in the elder PAS.

## CASE REPORTS/CASE HISTORIES

In a case report by Moorthy *et al*. “Levofloxacin-induced acute psychosis”[17] psychosis and not delirium is described for the patient though in their review they describe that Fluoroquinolones are an under-recognized cause of Delirium and hallucinations particularly with ciprofloxacin. Within 48 hours of stopping levofloxacin, repeat psychiatric evaluation revealed him to be alert and oriented with no further hallucinations.

It is indeed the review mentions delirium for ciprofloxacin and not levofloxacin.

Kumar *et al*. in “ECT and Clozapine Combination Producing delirium: A Case Report”[18] mention that simultaneous use of ECT and clozapine has been controversial and describe a case going into delirium following the combination and the delirium resolving on discontinuation of ECT. As a response to this in a letter to the editor Kurian *et al*. in their study “Combination of ECT and clozapine in drug-resistant schizophrenia”[19] report using a combination of clozapine and ECT in 3 men (21, 24 and 33 years of age) with schizophrenia resistant to treatment with conventional and atypical antipsychotic medications. They received 350-450 mg of clozapine per day and a course of 8-10 ECTs each. They did not have significant cognitive deficits or delirium and completed their courses of ECT. All the three patients showed a 40%-50% reduction in their Brief Psychiatric Rating Scale (BPRS) scores.

This clearly brings out the multifactorial nature of delirium on a case to case basis and need for better studies.

Pradeep in his case report on “Valproate monotherapy induced-delirium due to hyperammonemia: A report of three adult cases with different types of presentation”[20] makes a point that Valproate-induced delirium due to hyperammonemia (VIDH) is one of the rare and interesting adverse effects seen in patients on valproate therapy. In his three cases, he found that there was an elevated level of plasma ammonia in the cases during the delirious state and which decreased when valproate was discontinued. He also further rechallenges valproate in one of the cases, to prove that valproate caused the hyperammonemia and suggests that plasma ammonia levels should be monitored routinely in all cases of altered mental status in patients receiving valproate therapy Valproate-induced delirium may be mistaken for psychosis or worsening of mania leading to improper diagnosis and poor management.

Clearly, a specific delirium syndrome with cause and effect relationship and a caution for clinicians.

Rao *et al*. in a case series of “Self-injurious behavior: A clinical appraisal.”[21] describe in their analysis of SIB (Self Injurious Behavior) in the category of very severe and Isolated form of SIB a 30-year-old male with alcohol dependence of 10 year duration who stopped drinking suddenly and 3 days later he was fearful, shabbily dressed, tremulous, started talking as if he was conversing with some one and told that some people were chasing him. That night he slept poorly and next day started saying that something is stuck in his lower left incisor tooth and pulled out the tooth with his hand. Patient claimed amnesia for this event after he recovered. He was diagnosed to have delirium tremens.

Delirium tremens a form of delirium commonly seen in practice could also be dangerous to self.

Ustundag *et al*. report “A case of neuroleptic malignant syndrome induced by olanzapine in postpartum period”[22] from the Department of Emergency Medicine, Dicle University, Faculty of Medicine, Turkey say that Neuroleptic malignant syndrome (NMS) is a life-threatening condition that occurs as a result of dopaminergic receptor blockage in nigrostriatal pathways. The main clinical symptoms of this syndrome are hyperthermia, muscle rigidity, autonomic instability and delirium. In their case, patient was 20 years old and NMS has been seen on the 10^th^ day of the olanzapine treatment in the post partum period for a relapse of schizophrenia however with the early diagnosis and appropriate treatment, none of the deadly complications, such as cardiovascular collapse and renal or respiratory insufficiency, has been occurred.

Delirium symptoms could occur in NMS.

Similarly Pal *et al*. in “Psychiatric co-morbidity associated with pheniramine abuse and dependence”[23] present two case reports of toxic psychosis induced by diphenhydramine and prophenpyridamine, respectively; literature reports that in one study, about 44% (*n* = 43) of pheniramine-abusing patients developed psychosis/delirium while only 32% (*n* = 75) of patients using other antihistamines developed psychosis/delirium. However, their two case reports presented illustrate the occurrence of psychotic symptoms/disorders with massive pheniramine abuse/dependence which is in keeping with the literature but no delirium.

Nayyar in a case report of “Serotonin syndrome associated with sertraline, trazodone and tramadol abuse”[24] describes a 55-year-old white man on therapy for major depressive disorder with Zoloft and trazodone since one year developed sudden delirium, confusion, agitation, tremor, insomnia, diaphoresis, myoclonus, hyperreflexia in lower extremities, mydriasis, tachycardia, and low grade fever with the recent tramadol abuse secondary to worsening knee pain. He had been stable on this medication regimen for one year and tramadol was the probable culprit behind the adverse reaction.

Presumptive diagnosis of Serotonin syndrome was made and trazodone, sertraline, tramadol were discontinued.

Again delirium forming a part of a known syndrome but precipitated by the addition of Tramadol.

Ghosh *et al*. in “An analysis of six cases of acute intermittent porphyria (AIP)”[25] describe that among the six patients, four had abdominal pain, five had autonomic instability, all six had mental symptoms, three had depression, two came in delirium, and three had an episode of seizure. Acute intermittent porphyria (AIP) has a triad of symptoms (abdominal pain, neuropathy and mental changes) and porphobilinogen in the urine. Urinary porphobilinogen is a moderately sensitive test, both cases of delirium tested positive for AIP Mental disorders accompany attacks in 24% to 80% of cases and psychiatric symptoms can dominate the picture. Psychiatric manifestations include hysteria, anxiety, depression, phobias, psychosis, organic disorders, agitation, delirium and altered consciousness ranging from somnolence to coma and the most common neuropsychiatric manifestation reported are delirium and depression.

Delirium can be a manifestation manifestation of AIP.

Parkar *et al*. in “Is this ‘complicated’ opioid withdrawal?”[26] report on seven patients with opioid dependence admitted in their de-addiction centre for detoxification that developed convulsions and delirium during the withdrawal phase. After ruling out all other possible causes of these complications, opioid withdrawal seemed to emerge as the most likely explanation. This was an uncommon clinical complication during the withdrawal phase. Four of these patients developed delirium; the primary mode of consumption was inhalation of vapors formed from opium heated on an aluminum foil; three patients also had a history of intravenous use. One of these patients had a history of comorbid benzodiazepine dependence; the rest denied any history of benzodiazepine abuse or dependence. CT scan done on a delirious patient and electroencephalography of a patient with convulsions did not reveal any abnormality. All the patients recovered with no clinical evidence of any neurological sequelae. The opioid that all the patients consumed was the street variety, which is generally contaminated.

Complications such as convulsions and delirium are recognized in alcohol withdrawal. However, these have not been described as a feature of opioid withdrawal. It is most likely that the complications manifested in these patients were due to concurrent use of another substance such as alcohol or benzodiazepines. Another possibility is the presence of a contaminant, which they were unfortunately unable to confirm. Nonetheless, the potentially life-threatening nature of these complications warrants that patients with primarily opioid dependence be carefully monitored.

An unusual presentation for delirium but maybe the cause is in the adulterated opioid and may contain other ingredients.

## PRESIDENTIAL ADDRESSES AND ORATIONS

AB Ghosh delivering his presidential address at the 58^th^ ANCIPS on “Psychiatry in India: Need to focus on geriatric psychiatry”[27] emphasized on common psychiatric disorders in the elderly population and that the presentation of their psychiatric illnesses differ from that of adult patients with the same illnesses. The presentation of some physical illnesses of the elderly is also different. He pointed out that in this group, physical illness often presents with psychological symptoms and vice versa. Some common mistakes during diagnosis are unnoticed medical conditions such as dementia, delirium and depression.

IRS Reddy’s Presidential address “Making psychiatry a household word”[28] at the 59^th^ ANCIPS mentions that a way to eliminate stigma is to find causes and effective treatments for mental disorder, saying that history suggests this to be true. Neurosyphilis (General paresis of Insane) and dementia due to pellagra are illustrative of mental disorders for which stigma has receded. Similarly, when pellagra was traced to a nutrient deficiency and nutritional supplementation with niacin was introduced, the condition was eventually eradicated in the developed world. Pellagra’s victims with delirium had been placed in mental hospitals early in the 20^th^ century before its etiology was clarified.

Savita Malhotra in her DLN Murthy Rao oration in 2007 on “Acute and Transient Psychosis (ATP): A paradigmatic approach”[29] delineates the Acute and transient psychosis as a descriptive entity recognized only with the advent of ICD-10 in 1992, where it is included under psychotic disorder (F23) as a three-digit code. The key features that characterize the disorder are an acute (within two weeks) onset in all the cases; presence of typical syndromes which are described as rapidly changing, variable, polymorphic states and typical schizophrenic symptoms; evidence for associated acute stress in a substantial number of cases and complete recovery in most cases within two to three months. Apart from these diagnostic criteria, ICD-10 also provides diagnostic guidelines which include the exclusionary clauses such as absence of criteria for affective disorders, organic factors, substance misuse, provided to clearly separate these ATPs from affective disorders, delirium, substance-induced psychiatric syndromes.

## EDITORIALS, REVIEWS, INVITED ARTICLES

In a review article “The story of antipsychotics: Past and present,” Ramachandraiah CT, Subramanian N, Tancer M[30] mention about Haloperidol in the treatment of delirium with the first clinical studies being conducted by Bloch on patients with delirium tremens. It resulted in no significant sedation except for hypotension.

Haloperidol is clearly drug of choice and safe in treatment of Delirium.

Grover S, Bhateja G, Basu D. writing on “Pharmacoprophylaxis of alcohol dependence: Review and update Part I: Pharmacology”[31] mention while discussing Topiramate as an anticraving agent that there is one reported case of psychotic features, a case of delirium in a patient who overmedicated with 800 mg of topiramate and tranylcypromine sulphate (170 mg) combined with alcohol.

E. Mohandas and V. Rajmohan in their invited article “Frontotemporal dementia: An updated overview”[32] clearly state that behavior changes are the most common initial symptom of FTD (62%). About 10% of patients with FTD present with memory problems as a first symptom. Common behavioral symptoms in FTD include apathy (32%) and disinhibition (16%). Further clinical and pathological diagnostic criteria proposed by McKhann and colleagues facilitate easy recognition of the various FTD syndromes. The six proposed criteria are: (1) Early and progressive change in personality or language; (2) impairment in social or occupational functioning; (3) a gradual and progressive course; (4) exclusion of other causes; (5) presence of deficits in the absence of delirium; and (6) exclusion of psychiatric causes such as depression.

N. Kar in an invited article on “Behavioral and psychological symptoms of dementia and their management”[33] says that sometimes agitation, irritation and many other BPSD symptoms may be due to an underlying medical cause. The patient may not be able to communicate the pain and distress in words. Pacing and restlessness may be due to drug side-effects. Delirium is a common trigger for BPSD and the Management of BPSD involve psychological, behavioral, environmental, and pharmacological interventions. Treatment plan is individualized considering the need of the person, type of BPSD, previous response and pre-morbid experiences.

Delirium on dementia and dementia being unmasked after delirium is seen. Delirium may indeed be an early manifestation of dementia or risk factor for mortality in the aged.

RK Solanki, P Singh, and MK Swami in a review article on “Clozapine: Current perspective”,[34] mention case reports of clozapine-induced neuroleptic malignant syndrome (NMS) and suggest that typical NMS does occur with clozapine and that its incidence may be as common as with the classic neuroleptics. The features of clozapine-induced NMS may be somewhat different, with fewer extra pyramidal side effects and a lower rise in creatine kinase levels but potentially lethal, neuroleptic malignant-like syndrome that is marked by fever and delirium without muscle rigidity. Toxic delirium can occur in about 3% of patients.

T.S. Sathyanarayana Rao and K.S. Shaji in an editorial “Demographic aging: Implications for mental health”[35] suggest that since specialized services are possible only in general or teaching hospitals depending on the availability of trained manpower there is an obvious need to provide services in the primary care setting to benefit more people. At present, the primary care physicians do not come across many cases of dementia and are not involved in dementia care. However, many older people with delirium and depression seek the help of the primary care doctor and hence doctors in primary care should be able to identify and manage delirium, dementia and depression.

The 3 Ds of older people delirium, dementia and depression should be taught and trained for all physicians.

V. Reddy and C.R. Chandrasekhar in their review “Prevalence of Mental and Behavioral disorders in India: A meta-analysis”[36] analyzed 13 psychiatric epidemiological studies consisting of 33572 persons in 6550 families yielded an estimate prevalence rate of 58.2 per thousand population. The prevalence rate was Organic psychosis (prevalence rate 0.4), schizophrenia (2.7), affective disorders (12.3), mental retardation (6.9), epilepsy (4.4), neurotic disorders (20.7), alcohol/drug addiction (6.9; and miscellaneous group (3.9) were estimated. The prevalence of psychiatric disorders is 58.2 per thousand means that there are about 5.7 crore people suffering from some sort of psychiatric disturbance. Of this, four lakh people have organic psychosis.

There was no separate estimate for delirium in their analysis as organic psychosis may have included it.

## POSTER/PAPER PRESENTATION, VIEWPOINT, SYMPOSIA

J.K. Trivedi in a symposium on “Undergraduate psychiatric education in South Asian countries”[37] recommends that the objective of undergraduate psychiatric education should be to equip medical students with core psychiatric knowledge useful in daily medical practice and since Primary-care psychiatric problems, anxiety, depression are in abundance in primary-care clinics and medical wards where Psychiatry is not taught. This could lead to failure of undergraduate students to recognize common problems like anxiety and depression and hence the undergraduates may be taught the psychiatric manifestations of common physical disorders like delirium, which is found in abundance in medical and surgical wards.

Sahoo S, Manjunatha N, Sinha BN, Khess C. in their viewpoint article on “Why is alcohol excluded and opium included in NDPS act, 1985?”[38] raise the issue that there is a need for the reduction in the demand of drugs of addiction, both legal and illegal, which may otherwise lead to numerous health, family and societal consequences. To combat this, the Government of India formulated the Narcotic Drugs and Psychotropic Substances (NDPS) Act, 1985,[3] which provides the current framework for drug abuse control and sale in this country. Essentially, the Act deals with supply reduction activities of psychotropic substances namely, cannabis, cocaine and opium. However, the absence of alcohol in the list of psychotropic substances is surprising given the fact that mental health professionals consider alcohol to be a psychoactive substance leading to various social, legal, economic and medical complications ranging from gastritis to withdrawal seizures and delirium tremens.

Meera Narsimhan in a symposium on Geriatric psychiatry update spoke on Delirium: Diagnostic and treatment implications.[39] The incidence of delirium may be as high as 50% in hospitalized patients with dementia and that symptoms of delirium include a difficulty in coherent thinking, anxiety, restlessness, insomnia, disturbing dreams, and fleeting hallucination. Delirium is multifactorial and needs to be diagnosed appropriately and treated. Effective screening including a thorough physical examination, routine blood draws including RRR for syphilis, folate, Vitamin B 12 and TSH is requisite. Something as straightforward as a distended bladder or constipation, sensory deprivation, fever, infection, and the process referred to as “actively dying” may all be implicated. As with all symptoms, it is important to assess, intervene, and then reassess. Another important cause of delirium to screen for is polypharmacy. Discontinue any medications that are not needed, paying special attention to antidepressants, anticonvulsants, beta - blockers, antineoplastics and benzodiazepines. Although commonly used as a therapy for delirium, this class of drugs has been shown to worsen delirium.

Majority of the cases of delirium can be reversed. Using a combination of pharmacologic and non pharmacologic interventions will lead to the best clinical outcome.

Pharmacologic therapy for hyperactive delirium includes antipsychotics, such as haloperidol and chlorpromazine. Newer antipsychotic medications are also being used to treat delirium. Delirium with a high mortality rate needs to be accurately diagnosed and aggressively treated.

A comprehensive presentation on delirium.

Danivas V *et al*. in a poster presentation at ANCIPS 2009 on “Delirious Mania: A report of 2 cases from a psychiatric inpatient unit”[40] say what is also known as Bells Mania is a syndrome of excitement delirium and psychosis of acute onset. Delirium can confuse suggesting organic etiology. The two cases presented with disorientation, aggressive behavior, double incontinence, grandiose ideas and auditory hallucinations. Organic etiology was ruled out by investigations and ECT was instituted which resolved the delirium by 2^nd^ ECT and unmasked underlying Mania.

Delirium with a functional etiology stressing the need for careful analysis of delirium.

## CONCLUSIONS

The search on Acute Organic Brain Syndrome: Delirium yielded Clinical studies, case reports, reviews, symposia, orations, presidential addresses, paper/poster presentations, in the Indian Journal of Psychiatry. The annotation on Delirium would hopefully fulfill a need of providing the full range of data for Indian referencing. There is, however, a requirement of more number of articles in this area of Delirium publishing in the Indian Journal of Psychiatry rather than only in International journals which would go a long way in strengthening the quality of IJP and increasing its international appeal.

The annotation should also clearly provide a framework for a takeoff on further clinical research, training for detection and treatment of Delirium, an active participation by psychiatrists (CL Psychiatry) and an area of understanding of Brain function and pathology. Consultation Liaison Psychiatry may in future further contribute to an interest in this acute, under-recognized syndrome[41] and enable psychiatrists and physicians to manage this condition more effectively.
